# Effects of vitamin D supplementation on glucose metabolism and pregnancy outcomes in GDM: a systematic review and meta-analysis

**DOI:** 10.3389/fmed.2026.1743776

**Published:** 2026-02-09

**Authors:** Rong Luo, Huijing Wang, Yangli Cao

**Affiliations:** 1 Department of Obstetrics and Gynecology, Haikou Maternal and Child Health Hospital, Haikou, Hainan, China; 2 Department of Medical Genetics, Haikou Maternal and Child Health Hospital, Haikou, Hainan, China; 3 Department of Obstetrics, Haikou Maternal and Child Health Hospital, Haikou, Hainan, China

**Keywords:** gestational diabetes mellitus, glucose metabolism, meta-analysis, pregnancy outcomes, vitamin D

## Abstract

**Objective:**

This systematic review and meta-analysis aimed to evaluate the efficacy and safety of vitamin D supplementation on glucose metabolism and pregnancy outcomes in gestational diabetes mellitus (GDM).

**Methods:**

To achieve this, we searched Chinese and English databases (PubMed, EMBASE, Cochrane Library, China National Knowledge Infrastructure, Wanfang Med Online, and VipInfo Chinese Journal Service Platform) up to September 2024. Data were analyzed using Review Manager 5.3 and Stata 15.1, presenting continuous variables as standardized mean difference (SMD) and dichotomous variables as relative risk (RR), both with 95% confidence intervals (CI). The Cochrane tool assessed the risk of bias.

**Results:**

Twenty studies involving 1,737 patients were included. Meta-analysis showed that compared to placebo, vitamin D supplementation significantly reduced fasting glucose (SMD: −1.01, *p* = 0.0002), 2-h postprandial glucose (SMD: −0.89, *p* = 0.0002), insulin levels (SMD: −0.64, *p* < 0.0001), and insulin resistance (SMD: −0.91, *p* = 0.001). Furthermore, it was associated with lower incidences of cesarean delivery (RR: 0.68, *p* < 0.0001), forceps-assisted delivery (RR: 0.44, *p* = 0.02), preterm birth (RR: 0.28, *p* < 0.0001), postpartum hemorrhage (RR: 0.27, *p* = 0.01), fetal distress (RR: 0.17, *p* = 0.004), neonatal asphyxia (RR: 0.22, *p* = 0.006), macrosomia (RR: 0.34, *p* = 0.001), and neonatal hyperbilirubinemia (RR: 0.49, *p* = 0.001). No significant differences were found for amniotic fluid excess (RR: 0.46, *p* = 0.10) or pre-eclampsia (RR: 0.60, *p* = 0.38).

**Conclusion:**

Vitamin D supplementation may improve glucose metabolism and reduce adverse pregnancy outcomes in GDM patients. However, the findings should be interpreted with caution due to substantial heterogeneity among studies and the methodological limitations of the included trials. These exploratory results highlight the need for further rigorous research to confirm the effects.

## Introduction

Gestational diabetes mellitus (GDM) is a pregnancy complication characterized by the first detection or diagnosis of abnormal glucose metabolism during gestation, and it has long been associated with maternal and neonatal complications. The diagnostic criteria include a 75 g oral glucose tolerance test (OGTT) with fasting plasma glucose ≥5.1 mmol/L, 1-h glucose ≥10.0 mmol/L, or 2-h glucose ≥8.5 mmol/L (1). According to the 2021 International Diabetes Federation (IDF) Diabetes Atlas report, the standardized global prevalence of GDM is 14.0% (95% confidence interval 13.97–14.04%). Among regions, the Western Pacific has a prevalence of 14.7%, while Southeast Asia has a significantly higher rate of 20.8% (2). Previous studies have shown that GDM is one of the most typical complications of pregnancy, and women with GDM have an increased chance of preeclampsia, preterm labor, cesarean section, amniotic fluid overload, postpartum hemorrhage, and infection (3). In addition, GDM can cause respiratory distress syndrome, jaundice, hypocalcemia, and hypoglycemia in fetuses and newborns. Macrosomia can cause shoulder dystocia, neonatal ischemic–hypoxic encephalopathy, fractures, and even death (4). Although GDM usually subsides after delivery, it may have long-term health consequences. GDM can increase the mother’s risk of type 2 diabetes mellitus (T2DM), metabolic syndrome, and cardiovascular disease (CVD), as well as the child’s future risk of developing obesity and a significantly increased risk of T2DM (5, 6). Multiple studies have investigated risk factors associated with GDM. Pre-pregnancy overweight (BMI 25–29.9 kg/m^2^) and obesity (BMI ≥ 30 kg/m^2^) can increase the risk of GDM by approximately twofold and fourfold, respectively (adjusted OR 2.01 and 3.98) (7). The physiological elevation of pregnancy-related hormones (such as human placental lactogen, estrogen, and progesterone) directly contributes to its pathogenesis by exacerbating peripheral insulin resistance (1). Additionally, a meta-analysis on vitamin C intake suggests that antioxidant mechanisms may play a significant role in GDM, though high-quality randomized controlled trials (RCTs) data remain insufficient (8).

In recent years, the number of studies on GDM has been increasing, and some results have shown that vitamin D levels may be associated with glucose metabolism and pregnancy outcomes in patients with GDM (9). Vitamin D may influence maternal and fetal outcomes by affecting calcium absorption, parathyroid hormone expression, phosphate metabolism, and insulin-like growth factor regulation (9). Vitamin D supplementation in patients with GDM is a cost-effective public health strategy to minimize adverse maternal outcomes (9). The association between maternal vitamin D deficiency and GDM (OR 1.18, 95% CI 1.01–1.35) has been confirmed by a systematic review (10). Beyond micronutrient supplementation, broader maternal nutritional interventions have been recognized as a promising strategy to address the adverse outcomes associated with GDM (11). Given the established link between vitamin D deficiency and GDM risk, and the relative abundance of clinical trials, this meta-analysis focuses specifically on evaluating the role of vitamin D supplementation.

Previous studies by Yazdchi et al. (12) found that vitamin D supplementation in patients with GDM improved fasting blood glucose and Glycated Hemoglobin A1c (HbA1c), but no significant changes in fasting insulin or insulin resistance were observed between the two groups of patients treated with vitamin D versus placebo. Valizadeh et al. (13) found that vitamin D supplementation in GDM patients did not affect fasting blood glucose, fasting glucose, and insulin levels, or insulin resistance. However, Li et al. (14) found that supplementation with vitamins or minerals significantly improved glucose metabolism, such as fasting glucose, serum insulin, and insulin resistance in women with GDM, and also reduced inflammation and oxidative stress. However, as outlined above, evidence regarding the efficacy of vitamin D supplementation for improving glucose metabolism and pregnancy outcomes in GDM remains inconsistent. Thus, we performed this meta-analysis to comprehensively and systematically evaluate the efficacy and safety of vitamin D supplementation in improving glucose metabolism and pregnancy outcomes in patients with GDM.

## Methods

### Literature search strategy

This study adopted the Cochrane principles, and English and Chinese databases, such as PubMed, EMBASE, Cochrane Library, China National Knowledge Infrastructure (CNKI), Wanfang Med Online, and VipInfo Chinese Journal Service Platform databases, were searched to retrieve articles published from the date of the establishment of the databases to September 2024. Search terms included ‘gestational diabetes mellitus’ and ‘vitamin D’. Only Chinese and English language publications were included; studies in other languages were excluded.

#### Inclusion criteria

(i) Study type: RCTs; (ii) Study subjects: Patients with GDM; (iii) Interventions: The intervention group received supplementation with vitamin D, with no restrictions on specific dosage or duration; the control group received a placebo, no intervention, or conventional treatment; (iv) Outcome measures: At least one primary outcome was reported, such as glycemic control indicators or pregnancy outcomes; (v) Language and time: Publications in Chinese or English; publication date up to September 2024.

#### Exclusion criteria

(i) Non-randomized controlled studies, such as case–control, cohort studies, retrospective analyses, and animal experiments; (ii) Studies that did not supplement vitamin D alone or in combination, or did not report dosage and duration; (iii) Non-GDM patients; (iv) Inability to extract or estimate data for primary outcome measures; (v) Multiple reports from the same cohort or duplicate study results—only the most recent or complete publication was retained.

### Data extraction

Two researchers independently extracted the relevant information from the included studies, such as the author, year of publication, region, number of participants, treatments in the intervention and control groups, and primary outcome data. A third researcher checked the information and reviewed the data to ensure the accuracy of the information.

### Assessment of the quality of the literature

Two researchers evaluated the quality of the included studies based on the evaluation criteria recommended by the Cochrane Systematic Evaluation Guidance Manual (15). The tool examined the following components: (i) the generation of random allocation schemes; (ii) the concealment of allocation sequences; (iii) The implementation of blinding for all investigators and subjects; (iv) the implementation of blinding for outcome assessment; (v) the completeness of the data results; (vi) the selective reporting of results; and (vii) Other sources of bias. Each risk-of-bias level was categorized as low, high, or unclear, and the results are presented in different color blocks with the corresponding risk-of-bias maps. In the event of disagreement during the assessments, a third researcher made the final assessment.

### Statistical analysis

In this study, the continuous variables are presented as the standardized mean difference (SMD) and the corresponding 95% confidence interval (CI), while the dichotomous variables are presented as the relative risk (RR) and the corresponding 95% CI. The 95% CIs for all pooled estimates were calculated using inverse-variance weighted random-effects models. Study heterogeneity was assessed using the *I*^2^ statistic and the *χ*^2^ test, with an *I*^2^ value > 50% considered to indicate substantial heterogeneity. A subgroup analysis and sensitivity analysis were conducted if the heterogeneity was high. Publication bias was assessed using Begg’s test and Egger’s test. Forest and funnel plots were generated using Review Manager 5.3 software (The Cochrane Collaboration). The statistical analysis was performed using STATA 15.1 software (StataCorp, USA). A two-sided *p* value < 0.05 was considered statistically significant for the overall effect estimate.

## Results

### Literature search, study characteristics, and risk of bias

**Figure 1:**
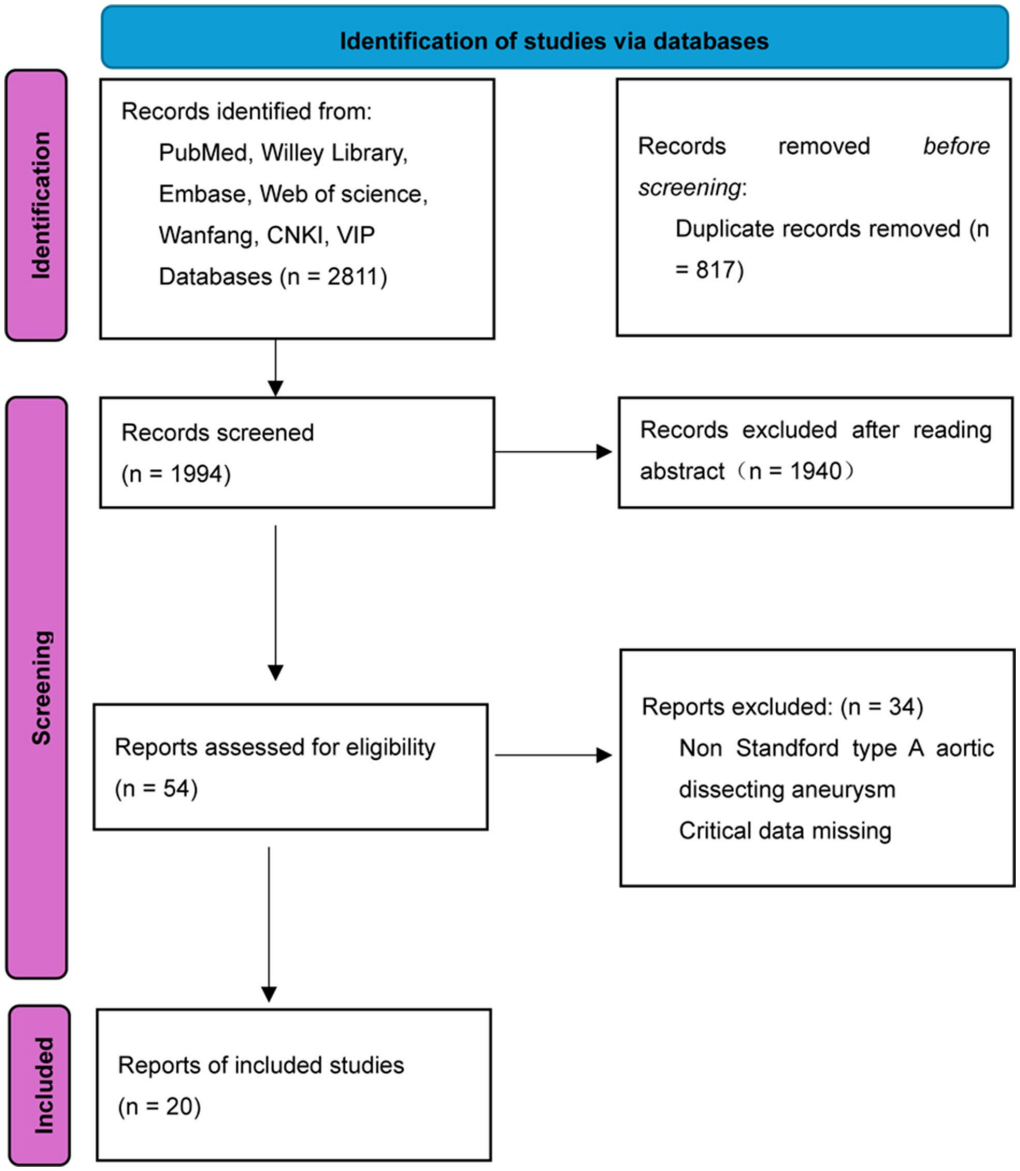
Flow chart of the included studies. This PRISMA flow diagram illustrates the study selection process, detailing the number of records identified, screened, assessed for eligibility, and included in the meta-analysis, as well as reasons for exclusions at each stage.

**Figure 2:**
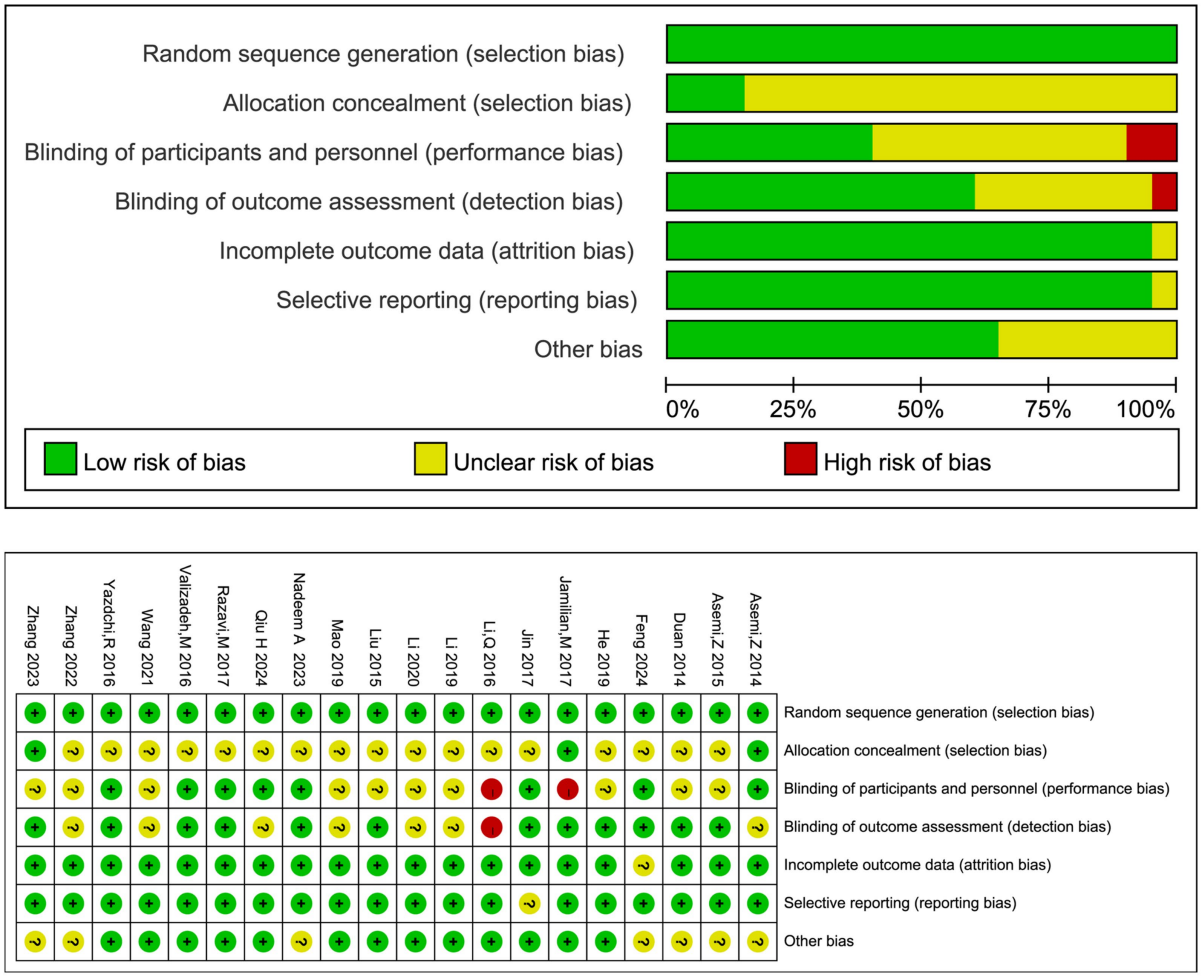
Risk of bias assessment for the included randomized controlled trials. Generated using the Cochrane Collaboration’s risk of bias tool (RevMan 5.3), this figure summarizes the methodological quality of the included studies across seven domains (e.g., randomization, blinding). The upper panel shows the proportion of studies with low, high, or unclear risk; the lower panel provides the detailed judgment for each study.

A total of 2,811 records were initially retrieved from the databases (1,736 in English and 1,075 in Chinese). After removing 817 duplicates, 1994 records remained for title and abstract screening. Following this screening, 1940 records were excluded, leaving 54 full-text articles assessed for eligibility. Of these, 34 were excluded for reasons such as non-conforming study design or missing critical data, resulting in the final inclusion of 20 RCTs in the meta-analysis. The study selection process is detailed in the PRISMA flow diagram (Figure 1). The baseline characteristics of the 20 included RCTs (12, 13, 16–33) and patients are presented in Table 1. The risk of bias assessment for included studies is presented in Figure 2. The majority of studies demonstrated low risk of bias, while two studies (18, 19) showed high risk. Both studies exhibited a high risk in random sequence generation due to a lack of blinding during allocation. Additionally, the study (19) presented a high risk in outcome assessment because blinding was not implemented during result evaluation.

**Table 1:** Baseline characteristics of the included studies.

Author	Country	Number of treatment	Number of control	Treatment	Control	Route of administration	Age of treatment group	Age of control group
Yazdchi, 2016 (12)	Iran	36	36	VitD_3_ 50,000 U/2 weeks for 2 months	Placebo	Oral	31.64 ± 4.40	32.11 ± 3.61
Valizadeh, 2016 (13)	Iran	42	42	Total 700,000 U	Blank control	Oral	32.0 ± 5.5	32.4 ± 4.7
Asemi, 2015 (16)	Iran	22	23	VitD_3_ 50,000 U twice (Before and day 21 intervention)	Placebo	Oral	31.1 ± 5.5	30.8 ± 6.2
Asemi, 2014 (17)	Iran	28	28	VitD_3_ 50,000 U (before and day 21 intervention) + Ca 1,000 mg /d for 6 weeks	Placebo	Oral	28.7 ± 6.0	30.8 ± 6.6
Jamilian, 2017 (18)	Iran	35	35	VitD_3_ 50,000 U/2 week	Placebo	Oral	31.5 ± 7.0	30.7 ± 4.1
Li, 2016 (19)	China	49	48	VitD_3_ 500 U + yogurt drink 100 mg, Twice daily	Yogurt drink	Oral	29.0 ± 5.3	28.3 ± 4.1
Razavi, 2017 (20)	Iran	30	30	VitD_3_ 50,000 U/2 week	Placebo	Oral	29.2 ± 3.4	29.9 ± 5.0
Nadeem, 2023 (21)	PAK	17	17	VD3 200,000 IU	Blank control	Intramuscular	25.24 ± 3.2	27.0 ± 1.7
Qiu, 2024 (22)	China	70	59	400 units of vitamin D3	Blank control	Oral	31.61 ± 5.10	30.00 ± 4.51
Duan, 2014 (23)	China	26	30	VitD_3_ 400 IU, twice daily	Blank control	Oral	28 ± 5	
He, 2019 (24)	China	30	30	VitD drops (D3) 800 IU/d	Blank control	Oral	30.2 ± 1.9	
Zhang, 2022 (25)	China	47	47	VitD 200 U–1,200 U/d	Blank control	Oral	29.24 ± 3.46	28.46 ± 3.38
Li, 2020 (26)	China	52	52	VitD 400 U–1,200 U/d	Blank control	Oral	28.93 ± 2.05	28.85 ± 2.01
Li, 2019 (27)	China	45	45	VitD 400 U–1,200 U/d	Blank control	Oral	29.65 ± 4.18	29.30 ± 4.23
Wang, 2021 (28)	China	92	92	VitD drops 400 IU–1,200 IU/d	Blank control	Oral	27.0 ± 3.4	27.6 ± 3.5
Li, 2015 (29)	China	44	41	VitD_3_ 7.5 mg intramuscular injection	Blank control	Intramuscular	36.0 ± 1.9	
Mao, 2019 (30)	China	59	59	VitD_3_ 400 U–1,200 U/d	Blank control	Oral	26.36 ± 3.34	25.86 ± 4.34
Jin, 2017 (31)	China	29	30	VitD soft capsule, 2,000 IU/d	Blank control	Oral	–	
Feng, 2024 (32)	China	30	30	VitD_3_ 400 IU, Twice daily	Blank control	Oral	26.39 ± 1.2	
Zhang, 2023 (33)	China	86	94	VitD_3_ 400 IU	Blank control	Oral	33.45 ± 4.62	

### Effects on glucose metabolism

**Figure 3:**
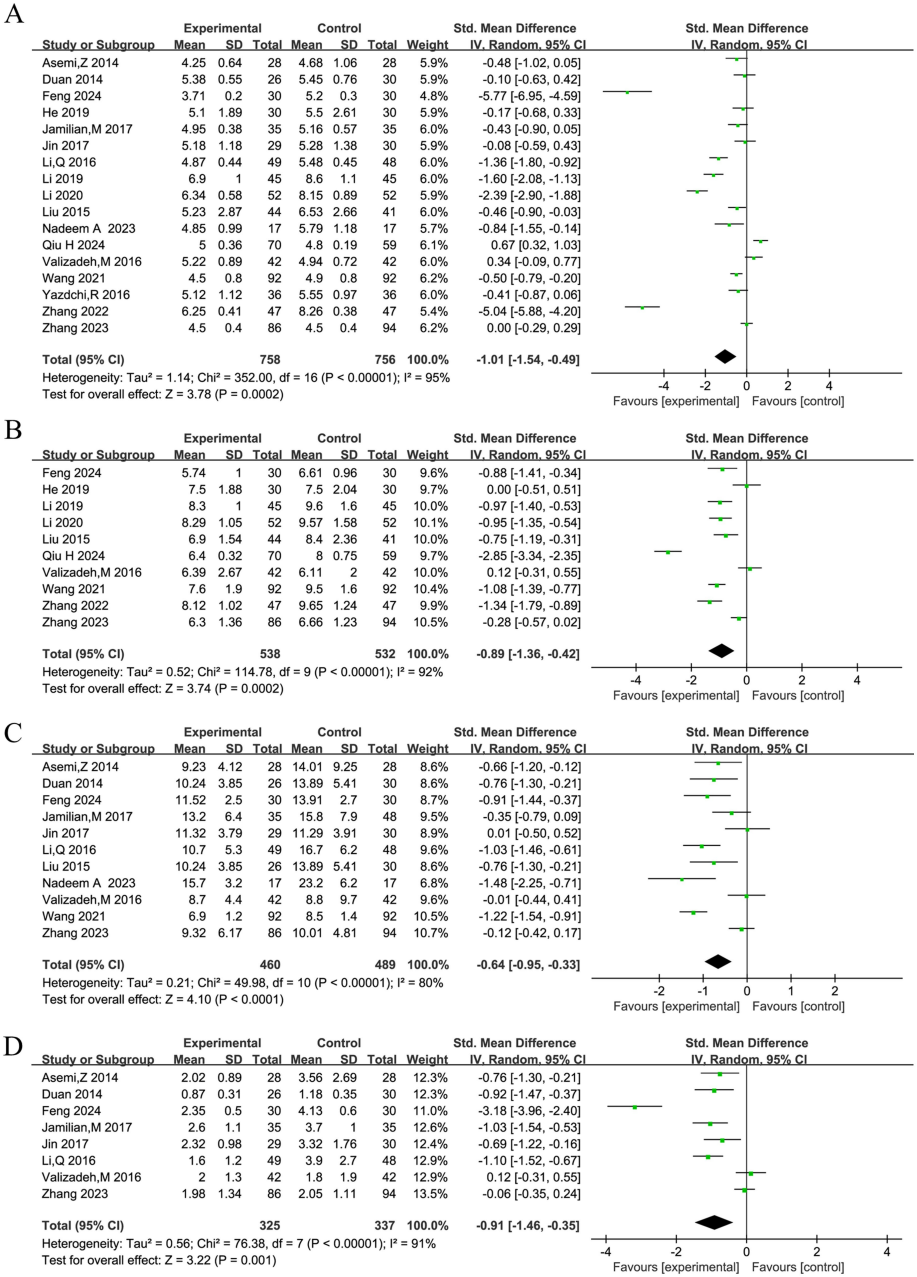
Forest plots of the effects of vitamin D supplementation on glycemic control parameters in women with GDM. Generated using Review Manager 5.3, these plots present the pooled standardized mean difference (SMD) and 95% CI for each outcome using a random-effects model **(A)** Fasting blood glucose (17 studies). **(B)** 2-h postprandial blood glucose (10 studies). **(C)** Insulin levels (11 studies). **(D)** Insulin resistance (8 studies).

Pooled analyses of four glycemic parameters consistently demonstrated beneficial effects of vitamin D supplementation (Figures 3A–D). A total of 17 studies reported fasting blood glucose. Due to significant heterogeneity (*I*^2^ = 95%), a random-effects model was used, showing a significant reduction with supplementation (SMD = −1.01, 95% CI: −1.54, −0.49, Figure 3A). Similarly, analysis of 10 studies on 2-h postprandial blood glucose (*I*^2^ = 92%) showed a significant reduction (SMD = −0.89, 95% CI: −1.36, −0.42; Figure 3B). Meta-analyses of insulin levels (11 studies; *I*^2^ = 80%) and insulin resistance (8 studies; *I*^2^ = 91%) also revealed significant improvements (SMD = −0.64, 95% CI: −0.95, −0.33, Figure 3C; and SMD = −0.91, 95% CI: −1.46, −0.35, Figure 3D, respectively). Begg’s and Egger’s tests indicated no significant publication bias for fasting blood glucose.

### Effects on maternal and neonatal outcomes

**Figure 4:**
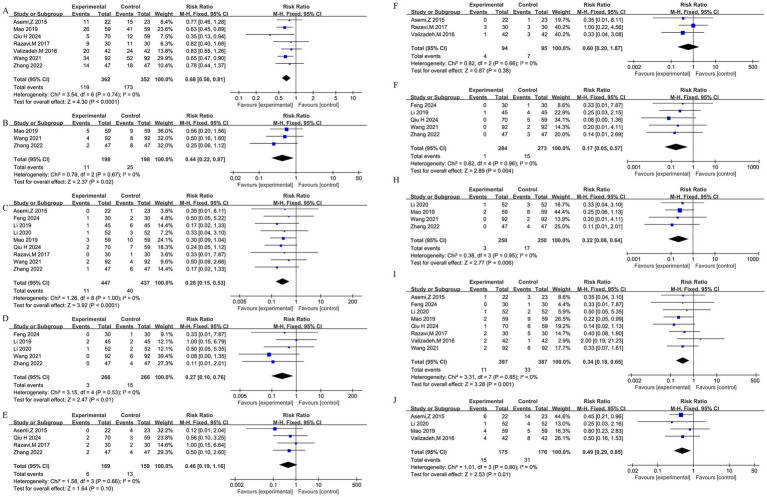
Forest plots of the effects of vitamin D supplementation on maternal and neonatal outcomes in women with GDM. Generated using Review Manager 5.3, these plots present the pooled risk ratio (RR) and 95% CI using fixed-effects models (for most outcomes due to low heterogeneity). **(A)** Cesarean section (7 studies). **(B)** Forceps-assisted delivery (3 studies). **(C)** Preterm birth (9 studies). **(D)** Postpartum hemorrhage (5 studies). **(E)** Polyhydramnios (4 studies). **(F)** Pre-eclampsia (3 studies). **(G)** Fetal distress (5 studies). **(H)** Neonatal asphyxia (4 studies). **(I)** Macrosomia (8 studies). **(J)** Neonatal hyperbilirubinemia (4 studies).

Vitamin D supplementation was associated with a reduced risk of multiple adverse pregnancy and neonatal outcomes, as detailed in Figures 4A–J. Fixed-effects models were applied due to low heterogeneity (*I*^2^ = 0% for most). For maternal outcomes, significant risk reductions were observed for cesarean section (seven studies: RR = 0.68, 95% CI: 0.58, 0.81; Figure 4A), forceps-assisted delivery (three studies: RR = 0.44, 95% CI: 0.22, 0.87; Figure 4B), preterm birth (nine studies: RR = 0.28, 95% CI: 0.15, 0.53; Figure 4C), and postpartum hemorrhage (five studies: RR = 0.27, 95% CI: 0.10, 0.76; Figure 4D). No significant effects were found for polyhydramnios (four studies: RR = 0.46, 95% CI: 0.19, 1.16; Figure 4E) or pre-eclampsia (three studies: RR = 0.60, 95% CI: 0.20, 1.87; Figure 4F). For neonatal outcomes, significant risk reductions were found for fetal distress (five studies: RR = 0.17, 95% CI: 0.05, 0.57; Figure 4G), neonatal asphyxia (four studies: RR = 0.22, 95% CI: 0.08, 0.64; Figure 4H), macrosomia (eight studies: RR = 0.34, 95% CI: 0.18, 0.65; Figure 4I), and neonatal hyperbilirubinemia (four studies: RR = 0.49, 95% CI: 0.29, 0.85; Figure 4J). Publication bias was not detected for cesarean section, preterm birth, or macrosomia.

### Sensitivity and subgroup analyses

A sensitivity analysis was conducted in the outcome indicators with high heterogeneity, such as fasting blood glucose, 2-h postprandial blood glucose, insulin level, and insulin resistance. The results showed that the combined effect values were largely similar before and after the removal of any studies, indicating that the results of this study were stable.

**Figure 5:**
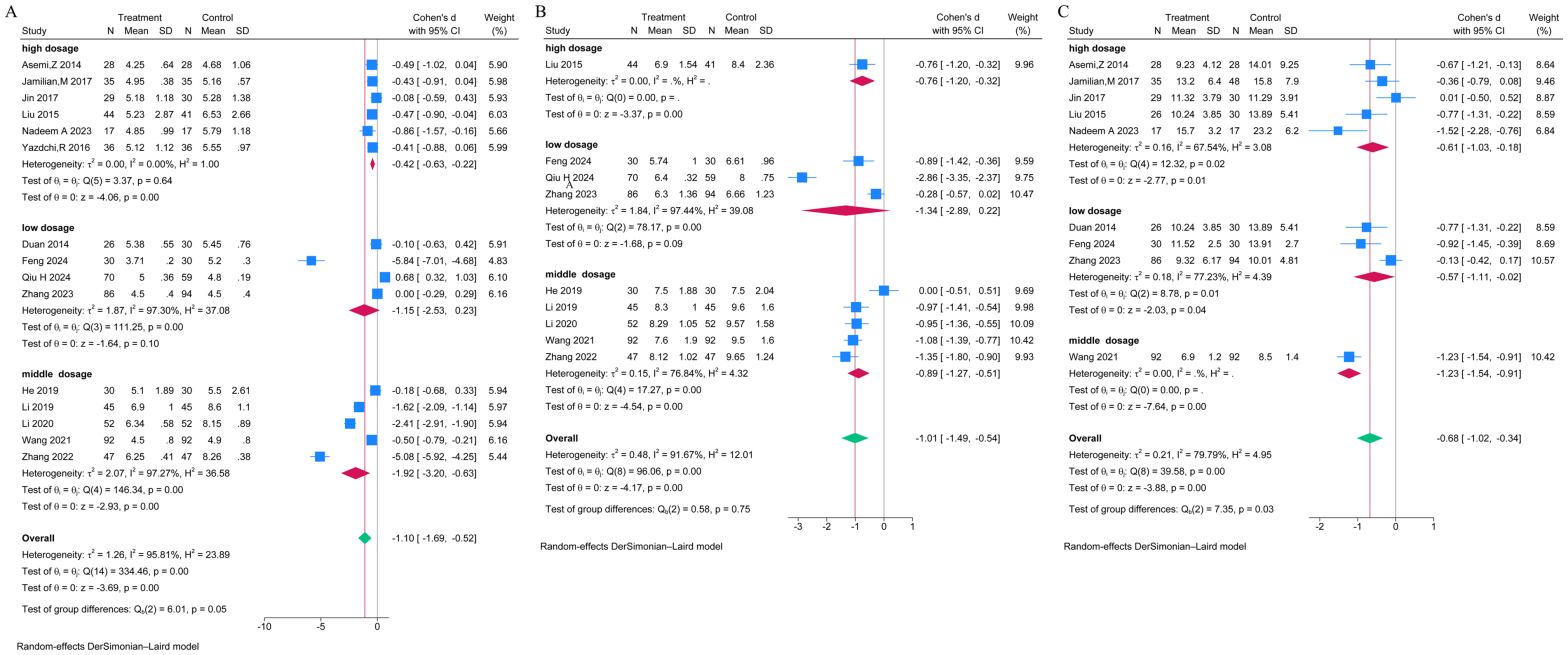
Subgroup analyses of glycemic outcomes by vitamin D dosage. Generated using Stata 15.1 software, these forest plots explore heterogeneity by stratifying studies into low- (<800 IU/day), moderate- (800–1,999 IU/day), and high-dose (≥2,000 IU/day) subgroups **(A)** Fasting blood glucose **(B)** 2-h postprandial blood glucose, **(C)** Insulin levels.

Subgroup analyses by vitamin D dose, presented in Figures 5A–C, were conducted to explore sources of heterogeneity. Doses were categorized into three groups: low-dose (<800 IU/day), moderate-dose (800–1,999 IU/day), and high-dose (≥2,000 IU/day or a single dose ≥200,000 IU). For fasting blood glucose, a marginally significant difference between subgroups was observed (*p* = 0.05), with the moderate-dose group showing the largest effect (SMD = −1.9), though heterogeneity remained high (Figure 5A). For 2-h postprandial glucose, no significant subgroup differences were found (*p* = 0.75), and heterogeneity persisted (Figure 5B). For insulin levels, significant subgroup differences were present (*p* = 0.03), but heterogeneity was not substantially reduced (Figure 5C). These analyses suggest that dose variation alone did not adequately explain the observed heterogeneity.

### Quality of evidence (GRADE)

Table 2 summarizes the GRADE assessment of the certainty of evidence. According to the GRADE assessment, the evidence for outcomes related to glucose and insulin metabolism (fasting blood glucose, 2-h postprandial glucose, insulin levels, and insulin resistance) was rated as low, primarily due to substantial heterogeneity and inconsistency in intervention protocols. Certain perinatal outcomes (e.g., cesarean section rate, preterm birth, fetal distress, and macrosomia) were rated as moderate, while most other perinatal and neonatal outcomes were rated as low to very low, limited by few studies, sparse events, or imprecision. The overall conclusions should be interpreted with caution, and more high-quality RCTs with consistent interventions are needed to improve the certainty of evidence.

**Table 2:** GRADE summary of findings: vitamin D supplementation versus placebo/no intervention for gestational diabetes mellitus.

Outcome	No. of studies (participants *n*)	Effect size (95% CI)	Heterogeneity *I*^2^	GRADE certainty	Reasons for downgrading
Fasting blood glucose	17 (1,514)	SMD = −1.01 (−1.54, −0.49)	95%	⊕⊕◯◯ Low	(1) Inconsistency ↓2: *I*^2^ = 95%; (2) Indirectness ↓1: High intervention heterogeneity.
2-h postprandial glucose	10 (1,070)	SMD = −0.89 (−1.36, −0.42)	92%	⊕⊕◯◯ Low	(1) Inconsistency ↓2: *I*^2^ = 92%; (2) Indirectness ↓1: Heterogeneity in intervention/concomitant care.
Insulin levels	11 (949)	SMD = −0.64 (−0.95, −0.33)	80%	⊕⊕◯◯ Low	(1) Inconsistency ↓1: *I*^2^ = 80%; (2) Indirectness ↓1: Differences in intervention type/concurrent therapy.
Insulin resistance	8 (662)	SMD = −0.91 (−1.46, −0.35)	91%	⊕⊕◯◯ Low	(1) Inconsistency ↓2: *I*^2^ = 91%; (2) Indirectness ↓1: Intervention/baseline status not stratified.
Cesarean section	7 (714)	RR = 0.68 (0.58, 0.81)	0%	⊕⊕⊕◯ Moderate	(1) Indirectness ↓1: Heterogeneous interventions & potential differences in obstetric management.
Forceps delivery	3 (396)	RR = 0.44 (0.22, 0.87)	0%	⊕⊕◯◯ Low	(1) Imprecision ↓1: Only 3 studies with sparse events; (2) Indirectness ↓1: Intervention/management heterogeneity.
Preterm birth	9 (884)	RR = 0.28 (0.15, 0.53)	0%	⊕⊕⊕◯ Moderate	(1) Indirectness ↓1: Uncontrolled differences in concurrent GDM management, gestational week at initiation, region, etc.
Postpartum hemorrhage	5 (532)	RR = 0.27 (0.10, 0.76)	0%	⊕⊕◯◯ Low	(1) Imprecision ↓1: Few events, wide CI; (2) Indirectness ↓1: Intervention/management heterogeneity.
Polyhydramnios	4 (328)	RR = 0.46 (0.19, 1.16)	0%	⊕◯◯◯ Very Low	(1) Imprecision ↓2: Wide & non-significant CI; (2) Indirectness ↓1: Intervention/management heterogeneity.
Pre-eclampsia	3 (189)	RR = 0.60 (0.20, 1.87)	0%	⊕◯◯◯ Very Low	(1) Imprecision ↓2: Very wide CI, uncertain result; (2) Indirectness ↓1: Intervention heterogeneity/uncontrolled concurrent management; Few studies.
Fetal distress	5 (557)	RR = 0.17 (0.05, 0.57)	0%	⊕⊕⊕◯ Moderate	(1) Indirectness ↓1: Potential influence of differences in obstetric management & intervention heterogeneity; Significant effect, *I*^2^ = 0%.
Neonatal asphyxia	4 (500)	RR = 0.22 (0.08, 0.64)	0%	⊕⊕◯◯ Low	(1) Imprecision ↓1: Sparse events, affecting reliability; (2) Indirectness ↓1: Intervention/management heterogeneity.
Macrosomia	8 (784)	RR = 0.34 (0.18, 0.65)	0%	⊕⊕⊕◯ Moderate	(1) Indirectness ↓1: Potential influence of concurrent factors like glycemic control & obstetric decisions; *I*^2^ = 0%, stable effect.
Neonatal hyperbilirubinemia	4 (351)	RR = 0.49 (0.29, 0.85)	0%	⊕⊕◯◯ Low	(1) Imprecision ↓1: Few studies/events; (2) Indirectness ↓1: Intervention/management heterogeneity.

CI, confidence interval; RR, risk ratio; SMD, standardized mean difference. Reasons for downgrading evidence: (1) Risk of bias: studies had methodological limitations. (2) Inconsistency: unexplained heterogeneity in results (*I*^2^ > 50%). (3) Indirectness: evidence differed from the population, intervention, comparator, or outcome of interest (e.g., heterogeneous interventions, unstratified baseline status). (4) Imprecision: wide confidence intervals or sparse data (few events/participants). (5) Publication bias: suspected or evident.

## Discussion

GDM is a serious complication of pregnancy with no clear pathogenesis. A clinical study found that the pathophysiological effect of GDM is transient, but it may have a significant effect on the health of the mother and fetus (34). Rodrigues et al. (35) reported that there is no moderate or high-quality evidence that vitamin D supplementation improves maternal glucose metabolism or the adverse maternal and neonatal outcomes associated with GDM compared to a placebo. Kron-Rodrigues et al. (36) found that vitamin D supplementation did not improve patients’ glucose metabolism indicators. Conversely, Wang et al. (37) found that vitamin D supplementation in women with GDM may improve glycemic control and reduce adverse maternal and infant outcomes. Further, a study of pregnant women showed that vitamin D supplementation may improve maternal insulin resistance and play a role in fetal growth (38).

The incidence of GDM is increasing with age (39). Previous studies have suggested that vitamins and minerals may be independent risk factors for the development of adverse events in patients with GDM. However, there is widespread controversy regarding whether supplementation with vitamin D improves glucose metabolism and pregnancy outcomes in patients with GDM, and there are inconsistent findings due to the presence of different factors, such as the sample size and vitamin D supplementation dose.

This study comprehensively analyzed the effects of vitamin D supplementation on glucose metabolism and pregnancy outcomes in GDM patients, and the results showed that supplementation with vitamin D improved glucose metabolism indicators among GDM patients, such as the fasting blood glucose level, 2-h postprandial blood glucose level, insulin level, and insulin resistance. Supplementation with vitamin D also reduced the risk of adverse outcomes, such as cesarean section, premature delivery, forceps-assisted delivery, postpartum hemorrhage, fetal distress, macrosomia, neonatal asphyxia, and hyperbilirubinemia. However, the pooled estimates for glycemic control were accompanied by high statistical heterogeneity (*I*^2^ > 80%), indicating substantial variability and suggesting that these findings should be interpreted as hypothesis-generating. Furthermore, potential adverse effects of high-dose supplementation were inadequately reported in the literature, particularly the toxicity concerns of long-term or high-dose vitamin D supplementation during pregnancy (e.g., hypercalcemia, etc.), which still warrant attention. Since vitamin D is a fat-soluble vitamin that can accumulate in the body, the relationship between dosage, efficacy, and safety requires further clarification. Therefore, future research should systematically evaluate the risks of bodily accumulation caused by high doses to ensure the safety of both pregnant women and fetuses.

This study had several limitations. First, although we included all available RCTs, the total number of studies and the sample sizes within them were still relatively limited, which may introduce bias and affect the precision of our estimates. Second, the extremely high heterogeneity (*I*^2^ > 80%) observed for key metabolic outcomes remains a major limitation, suggesting substantial unexplained clinical and methodological variability. Third, there was considerable diversity in the intervention protocols across studies, such as the type of vitamin D preparation, dosage, duration of supplementation, and the use of co-interventions (e.g., calcium, omega-3), which complicates the interpretation of the pooled effect. Fourth, important potential effect modifiers, such as baseline vitamin D status, gestational age at intervention initiation, and detailed concurrent therapies, could not be examined due to a lack of consistently reported data in the primary studies; this significantly limits the clinical applicability of our findings. Fifth, the included studies were conducted in a limited number of countries/regions, which may restrict the generalizability of the results to other populations with different genetic backgrounds, diets, or standard care practices.

To address the aforementioned limitations, we propose the following specific recommendations for future research. First, the dose range should be optimized in the study design to clarify the efficacy and safety of low, medium, and high doses (e.g., 400 IU/day, 2,000 IU/day, etc.), with dose subgroup analyses conducted. Future studies should strive for standardized and detailed reporting of supplementation protocols to facilitate robust dose–response evaluations in evidence synthesis. Second, future trials should clearly define the formulation and regimen of vitamin D supplementation and explore the effects of combining it with other nutrients (e.g., calcium) where biologically plausible. Third, attention should be paid to potential adverse effects of high-dose supplementation, particularly fluctuations in maternal blood calcium levels and impacts on fetal skeletal development. Fourth, it is recommended to conduct high-quality, multicenter, large-sample, long-term follow-up randomized controlled trials that include diverse populations (e.g., obese, advanced maternal age, or different ethnicities) to enhance the generalizability of the findings. Fifth, emerging “precision nutrition” strategies, such as genetic polymorphisms and microbiome analysis, could be incorporated to explore personalized supplementation regimens, providing more targeted evidence for clinical practice.

In summary, although this study supports that vitamin D supplementation may be associated with improved outcomes in GDM patients, the high heterogeneity and aforementioned limitations necessitate cautious interpretation of these pooled estimates. Furthermore, rigorously designed trials are needed to validate the optimal intervention strategy and assess long-term safety.

## Data Availability

The original contributions presented in the study are included in the article/supplementary material, further inquiries can be directed to the corresponding author.
